# Real-Time Accurate Apple Detection Based on Improved YOLOv8n in Complex Natural Environments

**DOI:** 10.3390/plants14030365

**Published:** 2025-01-25

**Authors:** Mingjie Wang, Fuzhong Li

**Affiliations:** 1 College of Agricultural Engineering, Shanxi Agricultural University, Jinzhong 030801, China; wangmj1@163.com; 2 College of Information Science and Engineering, Shanxi Agricultural University, Jinzhong 030801, China; 3 College of Software, Shanxi Agricultural University, Jinzhong 030801, China

**Keywords:** apple, lightweight architecture, real-time detection, Android deployment, YOLOv8

## Abstract

Efficient and accurate apple detection is crucial for the operation of apple-picking robots. To improve detection accuracy and speed, we propose a lightweight apple-detection model based on the YOLOv8n framework. The proposed model introduces a novel Self-Calibrated Coordinate (SCC) attention module, which enhances feature extraction, especially for partially occluded apples, by effectively capturing spatial and channel information. Additionally, we replace the C2f module within the YOLOv8n neck with a Partial Convolution Module improved with Reparameterization (PCMR), which accelerates detection, reduces redundant computations, and minimizes both parameter count and memory access during inference. To further optimize the model, we fuse multi-scale features from the second and third pyramid levels of the backbone architecture, achieving a lightweight design suitable for real-time detection. To address missed detections and misclassifications, Polynomial Loss (PolyLoss) is integrated, enhancing class discrimination for different apple subcategories. Compared to the original YOLOv8n, the improved model increases the mAP by 2.90% to 88.90% and improves the detection speed to 220 FPS, which is 30.55% faster. Additionally, it reduces the parameter count by 89.36% and the FLOPs by 2.47%. Experimental results demonstrate that the proposed model outperforms mainstream object-detection algorithms, including Faster R-CNN, RetinaNet, SSD, RT-DETR-R18, RT-DETR-R34, YOLOv5n, YOLOv6-N, YOLOv7-tiny, YOLOv8n, YOLOv9-T and YOLOv11n, in both mAP and detection speed. Notably, the improved model has been used to develop an Android application deployed on the iQOO Neo6 SE smartphone, achieving a 40 FPS detection speed, a 26.93% improvement over the corresponding deployment of YOLOv8n, enabling real-time apple detection. This study provides a valuable reference for designing efficient and lightweight detection models for resource-constrained apple-picking robots.

## 1. Introduction

Apple is one of the most widely cultivated fruits globally, with production reaching 95.83 million tons in 2022. China leads apple production, contributing 47.57 million tons [1]. In China, the apple industry provides a stable source of income for farmers and promotes the development of related processing industries. However, harvesting, a critical component of apple production, remains predominantly manual. As labor shortages intensify, the apple industry faces increasing challenges, driving a surge of interest in automating the harvesting process using mechanical picking devices.

Among the key enabling technologies for automated harvesting are visual algorithms, which have attracted growing research attention. Traditional machine vision methods often rely on manually extracted features such as texture, color, and shape for visual inspection [2,3,4]. However, these approaches are limited by human cognition, extracting insufficient features in complex orchard environments. As a result, they often suffer from low recognition accuracy and poor robustness, making them unsuitable for real-world fruit harvesting scenarios.

Deep learning has been widely adopted for agricultural perception tasks, attributed to its ability to automatically learn salient features from objects [5,6]. Compared to traditional machine learning algorithms, deep learning exhibits higher accuracy and stronger robustness for object detection in complex agricultural environments.

Object-detection models based on deep learning can be categorized into two main architectures: Convolutional Neural Networks (CNNs) and transformer-based structures. Models such as DETR [7] and RT-DETR [8], which utilize transformer structures, employ the self-attention mechanism to capture relationships between different regions in an image, enabling more accurate object detection. However, these transformer-based models often suffer from slow convergence during training and lengthy inference times, limiting their practicality in time-sensitive agricultural tasks.

CNN-based object-detection models primarily consist of SSD [9], Faster R-CNN [10], RetinaNet [11], and the YOLO series [12,13,14,15,16]. These CNN-based models typically converge more quickly and can achieve higher accuracy than transformer-based object-detection models when only a small data set is available for training.

Over the past few years, many scholars have applied CNN-based object-detection models to fruit detection [17,18]. For instance, Apolo-Apolo et al. [19] employed the two-stage Faster R-CNN [10] for citrus detection. This network was also utilized for multi-class apple occlusion detection in dense-foliage fruiting-wall trees, successfully identifying four classes of apples: non-occluded, leaf-occluded, branch/wire-occluded, and fruit-occluded, with average precisions of 90.9%, 89.9%, 85.8%, and 84.8%, respectively [20].

One-stage object-detection models, such as the YOLO series, offer faster detection speeds compared to two-stage networks while maintaining a balance between accuracy and speed, making them highly suitable for deployment in agricultural equipment. Wang et al. [21] developed an improved YOLOv8s for tomato detection and segmentation, enhancing accuracy across tomatoes at varying ripeness levels. Zhang et al. [22] achieved tomato visual detection and 3D pose estimation using the YOLOv5 framework. Hu et al. [23] integrated self-attention mechanisms from visual transformers into YOLOv7 and combined it with a multi-target tracking method using Kalman filtering and predictive motion trajectories. This integration improved mAP by 4% and the F1 score by 0.02 for apple orchard fruit detection and counting. Zhang et al. [24] designed a module combining multi-level channel and spatial attention mechanisms within the YOLOv3 framework for orange detection, achieving a mAP of 95.7%. Suo et al. [25] compared the performance of YOLOv3 and YOLOv4 for multi-category kiwifruit detection. They found that YOLOv4 achieved the highest accuracy for occlusion detection across five categories, reaching 91.9%.

Despite their effectiveness, many object-detection models face challenges such as high computational costs, large model sizes, and numerous parameters. To address these issues, researchers have explored lightweight improvements to existing models. Yu et al. [26] proposed the SOD-YOLOv5n model for winter jujube detection, improving accuracy by 2.40% while reducing the model size by 16.51% through structural adjustments to YOLOv5n. Lu et al. [27] improved the YOLOv5s model for green citrus detection in real environments by enhancing spatial and channel representations through feature weighting and fusing local and global information. This approach reduced the model size by 8.82% and the parameter count by 9.6% while improving accuracy by 1.5%. Zhao et al. [28] introduced a lightweight YOLO-GP architecture for the simultaneous detection of grapes and their picking points. By employing ghosting bottlenecks in the model architecture, they reduced the model’s parameter count by 10% compared to YOLOv4, achieving a mAP of 93.27%. Wang et al. [29] enhanced the YOLOv5s model for lychee recognition by introducing an attention mechanism and optimizing the underlying feature extractor. These modifications resulted in a 3.5% increase in mAP and a 62.77% reduction in model size.

While these methods effectively reduce model size and parameter count, they do not always lead to a direct reduction in detection latency. Improvements that reduce the parameter count and computational requirements may inadvertently increase memory access, which can slow down detection processes [30], thereby contradicting the initial purpose of lightweight improvements.

Researchers have conducted extensive studies on apple detection using deep learning-based algorithms. Wu et al. [31] proposed a lightweight apple-detection model, DNE-YOLO, based on YOLOv8. The model’s attention to apples was enhanced by introducing the CBAM attention mechanism, and the number of parameters was reduced using GSConv. Experimental results show that DNE-YOLO achieves an average accuracy of 94.3%. Wang et al. [32] improved the detection accuracy of small targets, such as apple fruits, by enhancing the RFA module, the DFP module, and the Soft–NMS algorithm, and incorporating them into YOLOv5s. Their model showed improvements of 3.6%, 6.8%, and 6.1% in precision, recall, and mAP, respectively. To enhance the detection precision of occluded apples, Wu et al. [33] introduced the SPD-Conv module into YOLOv8n. They also integrated the GAM global attention mechanism to improve the recognition of occluded targets, and optimized target frame regression using the Wise–IoU loss function. Experimental results demonstrated a detection accuracy of 75.9% and a detection speed of 44.37 FPS. Fu et al. [34] further improved detection by introducing the Diverse Branch Block (DBB), the SE attention mechanism, and the proposed Normalized Wasserstein Distance (NWD) loss function into YOLOv10. This led to improvements of 3.1%, 2.2%, and 3.0% in precision, recall, and average precision, respectively, resulting in a final accuracy of 89.3%, recall of 89.8%, and mAP of 92.8%. While these methods have significantly improved apple-detection accuracy, they are primarily designed for desktop computers. Research on apple-detection models optimized for mobile device deployment remains limited.

To address these challenges, this study proposes a novel apple-detection model based on a lightweight YOLOv8n architecture. The proposed model enhances detection speed and accuracy in orchard environments, making it particularly suitable for resource-constrained devices.

The primary contributions of this paper are as follows:

(1) An SCC attention module is developed to enhance the YOLOv8n model’s ability to detect occluded apples by effectively extracting relevant features. The performance of this module is rigorously compared with other attention mechanisms.
(2) A PCMR module is proposed to replace the C2f module in the YOLOv8n neck, reducing redundant computations, parameter count, and memory access during forward prediction.
(3) Features from the second and third levels of the backbone architecture are used for multi-scale information fusion, reducing computational costs and improving detection speed. Additionally, PolyLoss is used instead of cross-entropy loss to better adapt the model to apple-detection tasks, significantly reducing the misidentification rate for subcategories.
(4) The robustness of the proposed model is evaluated in unstructured orchard environments, and its performance is compared with other representative object-detection models. Furthermore, an Android application is developed to deploy the model on a smartphone, enabling real-time apple detection in an orchard environment.

## 2. Materials and Methods

### 2.1. Preparation of the Data Set

**Figure 1:**
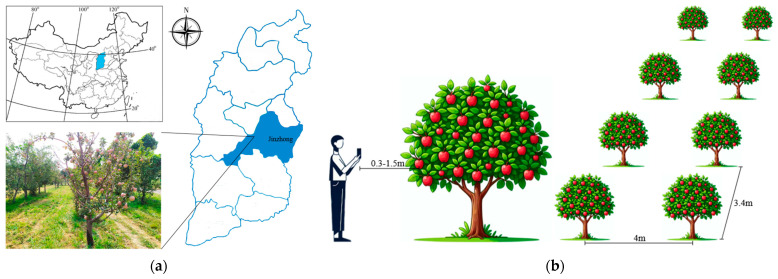
Schematic diagram of apple image acquisition. (**a**) Apple data-acquisition area; (**b**) Apple data set acquisition.

**Figure 2:**
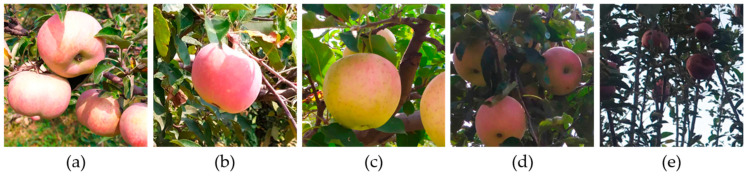
Sample of apples collected under different lighting conditions. (**a**) Direct light; (**b**) Side light; (**c**) Diffuse light; (**d**) Backlight; (**e**) Low light.

The images used in this study were collected from an apple orchard located in Taigu District, Jinzhong City, Shanxi Province, as shown in Figure 1a. The spacing between rows of apple trees in the orchard was 4 m, and the distance between apple trees in a row was 3.4 m. Images were collected in September 2019 and September 2022, specifically during the periods of 10:00 a.m. to 12:00 p.m. and 4:00 p.m. to 7:00 p.m. The acquisition device was a Redmi Note 7 smartphone, and the distance of the smartphone from the apples was within the range of 0.3–1.5 m, as depicted in Figure 1b. The images were acquired under various lighting conditions, including direct light, side light, diffuse light, backlight, and low light. Notably, some apples within these images were partially occluded by branches, leaves, or adjacent apples. To accelerate the training process of the YOLOv8n model, the images were compressed to a resolution of 640×640 pixels, totaling 1215 images, some of which are shown in Figure 2.

**Figure 3:**
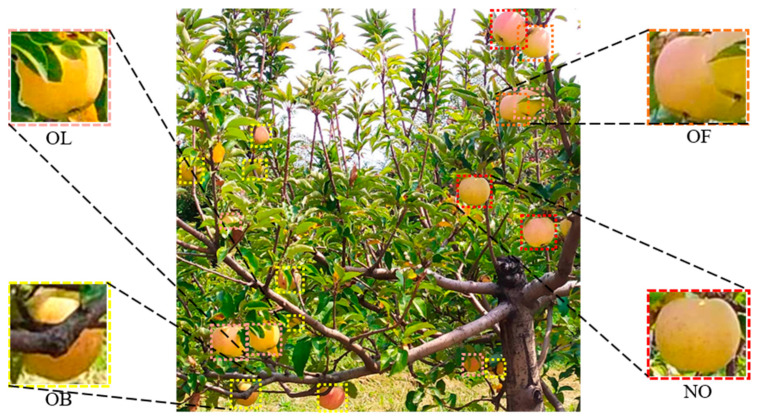
Display of apple data set annotation results. NO, OL, OF, and OB denote apples with no occlusion, occluded by leaves, occluded by other fruits, and occluded by branches, respectively.

The apples in the collected images were labeled with their locations and categories using the LabelImg 1.8.1 software. The labeling process involved marking rectangular boxes around the periphery of each visible apple in the collected images, and the category aspect considered the different impacts of occlusion by branches, leaves, or other apples on the picking robot [20]. The apples were classified into four categories: NO (no occlusion), OL (occluded by leaves), OF (occluded by other fruits), and OB (occluded by branches), as illustrated in Figure 3. It is worth noting that if more than one type of occlusion occurred for the same apple, the apple was labeled as OB if the occlusion type included branch occlusion; otherwise, the apple was labeled as OF. The labeled images were divided into three sets: training set, validation set, and test set, with a 7:1:2 ratio. The number of images in the training set was 851, and the number of fruits that belonged to each category (NO, OL, OF, and OB) were 4969, 3190, 1069, and 2808, respectively. The number of images in the validation set was 121, and the number of fruits containing NO, OL, OF, and OB were 641, 322, 171, and 429, respectively. The number of images in the test set was 243, and the number of fruits containing NO, OL, OF, and OB were 1191, 870, 343, and 705, respectively.

**Figure 4:**
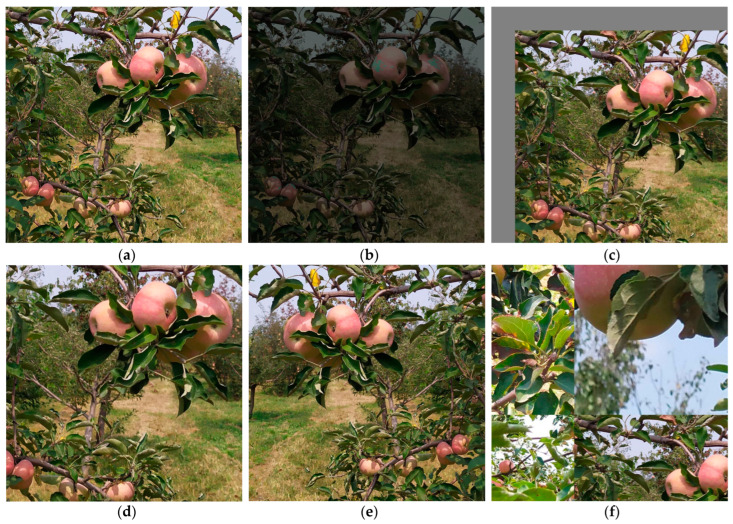
Image data augmentation. (**a**) Original image; (**b**) Random HSV adjust; (**c**) Random translation transformations; (**d**) Random Scaling; (**e**) Image flipping; (**f**) Random mosaic.

To prevent overfitting and enhance the model’s robustness, tools such as OpenCV were used to perform five types of data augmentation randomly on the images in the training set, including adjusting the HSV color space, translation transformations, scaling, image flipping, and mosaic operations. The maximum adjustment ratios for Hue, Saturation, and Value in the HSV color space were 0.015, 0.5, and 0.3, respectively; the maximum ratios for image translation and scaling were 0.1 and 0.4, respectively; the probability of image left-right flipping was 0.5; and four images were randomly selected for mosaic enhancement. Samples of the data augmentation are shown in Figure 4. It is worth noting that no data augmentation technique was applied to the validation and test sets. After employing data augmentation, the number of images in the training set increased to 2310, and the number of fruits containing NO, OL, OF, and OB were 11,502, 6767, 2518, and 5631, respectively. The detailed composition of the data set is shown in Table 1.

### 2.2. Overview of the YOLOv8n Model

**Figure 5:**
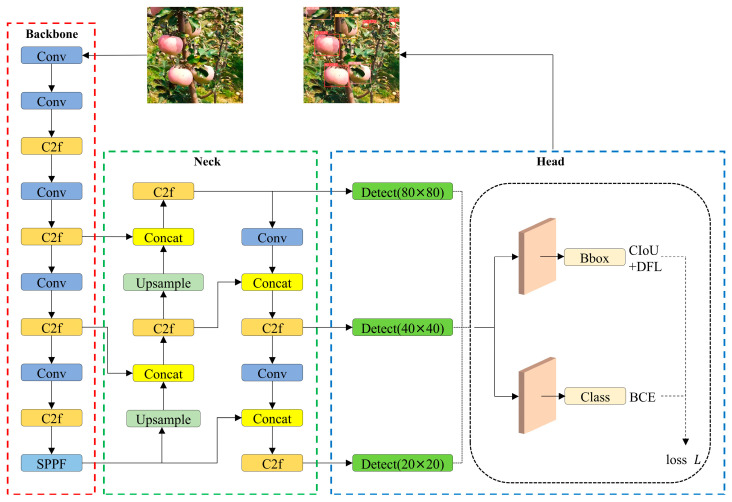
Structure of the YOLOv8n model.

YOLOv8 is an advanced one-stage object-detection model that predicts both the category and location of objects directly within images. It incorporates design ideas from ELAN (Efficient Layer Aggregation Network) in YOLOv7 and the decoupled head from YOLOv6, building on the foundation of YOLOv5. Furthermore, YOLOv8 introduces a novel backbone architecture and an innovative anchor-free detection head. The YOLOv8 family comprises five sub-models: YOLOv8n, YOLOv8s, YOLOv8m, YOLOv8l, and YOLOv8x. To achieve faster apple detection with a smaller model size and lower computational requirements, YOLOv8n was selected as the benchmark model. The structure of YOLOv8n consists of four parts: the backbone, the neck, the head, and the loss function, as illustrated in Figure 5.

The backbone network in YOLOv8 extracts features from the image, benefiting from the C2f module, which contains more skip connections compared to YOLOv5, leading to a richer gradient flow. The neck network integrates the C2f module into the Path Aggregation Network (PANet) structure, maintaining a lightweight design while effectively fusing multi-scale features from the backbone to improve model performance. The head network includes classification and regression branches that process the features from the neck network to output vectors representing object categories and bounding box locations. Notably, this is accomplished without predefined anchors, thereby reducing the time required for non-maximum suppression. The loss function is composed of classification loss and regression loss. Specifically, the regression loss is composed of Distribution Focal Loss (DFL) and Complete IoU (CIOU) Loss, while Binary Cross Entropy (BCE) Loss is employed for the classification loss. The total loss is the weighted sum of these individual losses.

### 2.3. The Improved YOLOv8n Model

**Figure 6:**
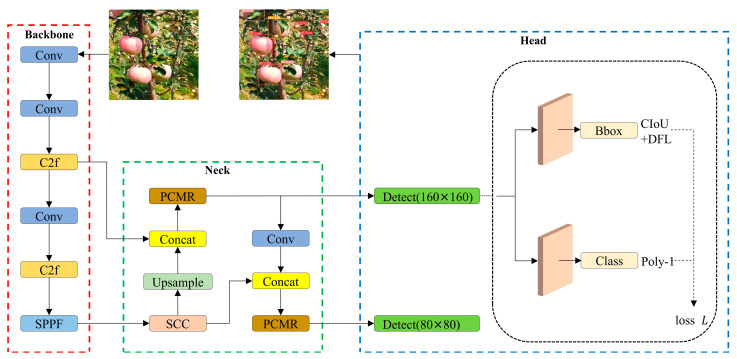
Improved YOLOv8n model structure.

To enhance the speed, accuracy, and efficiency of apple detection, and to make the model more suitable for deployment on apple-picking robots with limited computational resources, we improved the YOLOv8n model. The structure of the improved model is depicted in Figure 6.

The specific improvements to the YOLOv8n model include: (1) integrating SCC attention to enhance apple-detection accuracy; (2) replacing the neck’s C2f module with the PCMR module to increase detection speed; (3) substituting the cross-entropy loss function with PolyLoss for better apple subcategory recognition; and (4) employing the backbone’s P2 and P3 layers for multi-scale feature fusion (instead of the default P3, P4, and P5 layers) to improve the detection of smaller objects.

#### 2.3.1. Self-Calibrated Coordinate Attention

**Figure 7:**
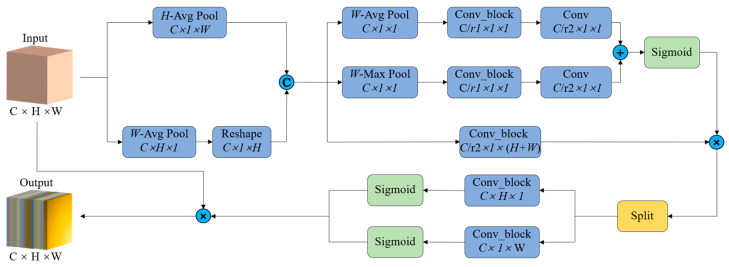
SCC attention module structure. The symbols C, ×, and + within the blue circle denote the operations of concatenation, element-wise multiplication, and element-wise addition, respectively.

The presence of occluded apples can reduce the model’s accuracy. To improve detection accuracy under such conditions, we designed a self-calibrated coordinate (SCC) attention module, inspired by the coordinate attention (CA) module [35]. The SCC attention module adaptively performs long-range spatial and inter-channel dependent calibration operations around each spatial location. This design enables more efficient extraction of both location and channel features, thereby enhancing the model’s ability to recognize partially occluded apples. The overall architecture of the SCC attention module is illustrated in Figure 7.

Specifically, given the feature maps x from the previous layer, pooling operations are applied to each channel along the horizontal and vertical coordinate directions, respectively. The output can be expressed as follows:

(1) $$z^{h}(h)=\frac{1}{W}\sum_{0\leq i\leq W}x(h,i)$$

(2) $$z^{w}(w)=\frac{1}{H}\sum_{0\leq j\leq H}x(j,w)$$

where W and H denote the width and height of the feature maps, respectively. Both transformations preserve precise location information along one spatial direction, enabling the network to accurately localize the apple’s position. The feature maps aggregated through vertical pooling, as defined in Equation (2), are rotated and then concatenated with the feature maps produced by horizontal pooling, as per Equation (1), to generate orientation-aware feature maps:

(3) $$z=[z^{h},z^{w^{\prime }}]$$

where [−,−] denotes the concatenation operation along the spatial dimension, and $z^{w^{\prime }}$ is obtained by rotating $z^{w}$. The orientation-aware feature maps serve as inputs to the self-calibration operation for subsequent processing.

The self-calibration operation is performed as follows: First, each channel is encoded using both average pooling and max pooling to capture global channel information. Thereafter, the pooled outcomes are fed into two shared 1 × 1 convolutional layers, denoted as $F_{1}$ and $F_{2}$, and the outputs from these layers are summed. Subsequently, the sum is processed by a sigmoid activation function to obtain the self-calibration weights:

(4) $$M=\sigma (F_{2}(F_{1}(AvgPool(z)))+F_{2}(F_{1}(MaxPool(z))))$$

where σ denotes the sigmoid activation function. The self-calibration weights M are subsequently multiplied element by element with the feature maps obtained by applying a 1 × 1 convolution operation $F_{3}$ to the feature z, completing the self-calibration operation as follows:

(5) $$y=M\cdot F_{3}(z)$$

The feature maps y from the self-calibration operation are divided along the spatial dimension into two tensors, $y^{h}$ and $y^{w}$. These tensors are then converted into $k^{h}$ and $k^{w}$, matching the number of channels in the input x, through two 1 × 1 convolution operations denoted as $F_{h}$ and $F_{w}$, respectively. This process is described as follows:

(6) $$k^{h}=\sigma (F_{h}(y^{h}))$$

(7) $$k^{w}=\sigma (F_{w}(y^{w}))$$

where $k^{h}$ and $k^{w}$ denote the attentional weights along the horizontal and vertical directions, respectively. Finally, the feature maps from the self-calibrated attention module are obtained, calculated as:

(8) $$y(i,j)=x\times k^{h}\times k^{w}$$

#### 2.3.2. Partial Convolution Module Improved with Reparameterization

**Figure 8:**
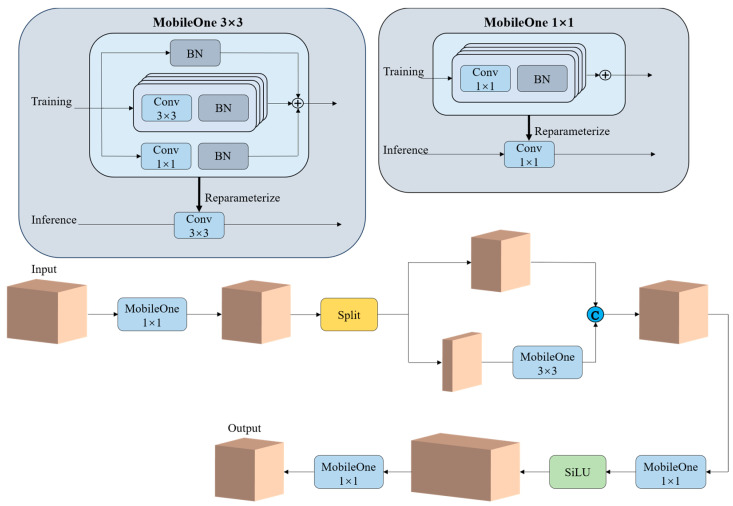
Structure of PCMR module.

To make the model lightweight and improve forward prediction speed, many studies have concentrated on minimizing FLOPs and parameter count. However, these metrics do not correlate well with latency, primarily due to frequent memory access by operators, which results in low floating-point operations per second (FLOPS) [30]. Moreover, operations independent of parameters, such as skip connections or branches, also incur significant memory access costs. Inspired by FasterNet [30] and MobileOne [36], we proposed the partial convolution module improved with reparameterization (PCMR), as shown in Figure 8. The PCMR incorporates partial convolution and reparametrizable branches into the PANet structure to more efficiently aggregate features of apples. This design minimizes redundant computations, reduces parameter count, and decreases memory access during forward prediction.

Specifically, input features are fused using pointwise convolutions (PWConv) to integrate information across different channels while compressing the number of channels to be equal to the output channel count of C2f at the same position in YOLOv8n. By leveraging the redundancy in feature maps, standard convolution (Conv) is selectively applied to a subset of input channels, thereby optimizing computational costs.

When only a quarter of the channels are subjected to standard convolution, the FLOPs for partial convolution are reduced to one-16th of those required for a standard convolution, and the memory access requirements are decreased to one-fourth of those required by a standard convolution [30].

Finally, the remaining channels, along with the output channels from the partial convolution, are fed into two consecutive PWConv layers, enabling features to propagate across all channels.

To effectively extract spatial features, we introduced re-parameterizable skip connections with batch normalization (BN), along with trivially overparameterized branches, into the convolutional layers of the PCMR. During inference, a re-parameterization process eliminates these branches [36]. Specifically, each BN layer is folded into its preceding convolutional layer, and skip connections are treated as 1 × 1 convolutions. In modules with different kernel sizes, smaller convolution kernels are zero-padded to match the larger kernel size. Ultimately, a final convolution kernel is obtained by summing multiple kernels of the same size.

As a result, during inference, the convolutional layers within the PCMR adopt an architecture without branches, eliminating additional latency costs associated with branched structures.

#### 2.3.3. Polynomial Loss

YOLOv8n employs the cross-entropy loss function for object classification, achieving high classification accuracy. However, for the specialized task of apple classification, cross-entropy loss may not fully address the unique challenges posed by this data set. To enhance classification performance for apples, we introduced polynomial loss (PolyLoss) [37] as a replacement for the cross-entropy loss function in YOLOv8n for apple classification. PolyLoss allows for flexible adjustment of the importance of different polynomial bases based on the specific task and data set, thereby optimizing classification performance for apples.

PolyLoss can be represented by Equation (9):

(9) $$L_{Poly}=\alpha_{1}(1-P_{t})+\alpha_{2}{\left(1-P_{t}\right)}^{2}+\ldots +\alpha_{N}{\left(1-P_{t}\right)}^{N}+\ldots =\sum_{j=1}^{\infty }\alpha_{j}{\left(1-P_{t}\right)}^{j}$$

where $\alpha_{j}\in {\mathbb{R}}^{+}$ represents the polynomial coefficients, and $P_{t}$ denotes the model’s predicted probability for the target ground-truth class. By applying Taylor expansion, the cross-entropy loss can be decomposed into a series of weighted polynomial bases, as shown in Equation (10), which can be viewed as a special case of PolyLoss, where $\alpha_{j}=1/j$ for all j.

(10) $$L_{\mathrm{CE}}=-\log(P_{t})=\sum_{j=1}^{\infty }1/j{\left(1-P_{t}\right)}^{j}=(1-P_{t})+1/2{\left(1-P_{t}\right)}^{2}\ldots$$

PolyLoss enables customization of loss functions for different data sets by adjusting the polynomial coefficients $\alpha_{j}$. However, due to the complexity involved, tuning all polynomial coefficients is impractical. Therefore, only the first N coefficients in the cross-entropy loss are perturbed [37], as expressed in Equation (11):

(11) $$\begin{array}{l} L_{Poly-N}=\underset{perturbed\;by\;\varepsilon_{j}}{\underset{︸}{(\varepsilon_{1}+1)(1-P_{t})+\ldots +(\varepsilon_{N}+1/N){\left(1-P_{t}\right)}^{N}}}+\ldots \\ =-log(P_{t})+\sum_{j=1}^{N}\varepsilon_{j}{\left(1-P_{t}\right)}^{j} \end{array}$$

where N represents the number of leading coefficients to be tuned and $\varepsilon_{j}\in [-1/j,\infty )$ is the perturbation term. Leng found that setting N=1, which modifies only the first polynomial coefficient, significantly improved classification accuracy [37]. Therefore, following the recommendation from the PolyLoss paper [37], we set N=1, denoted as $L_{Poly-1}$, as expressed in Equation (12):

(12) $$L_{Poly-1}=(1+\varepsilon_{1})(1-P_{t})+1/2{\left(1-P_{t}\right)}^{2}+\ldots =-log(P_{t})+\varepsilon_{1}(1-P_{t})$$

#### 2.3.4. Lightweight Feature Fusion Networks

YOLOv8n defaults to extracting features from layers p3, p4, and p5 of the backbone architecture as inputs for the neck networks, achieving a trade-off between detection accuracy and speed on the COCO data set. However, the variance in target object sizes in our apple data set is less pronounced compared to that in the COCO data set. Consequently, the default combination of feature layers may not be optimal for our apple data set. Therefore, we conducted experiments to identify a more suitable combination of feature layers for our apple data set.

### 2.4. Model Quantification and Deployment

To evaluate the improved YOLOv8n model’s performance on mobile devices with limited computational resources, we developed an Android-based apple-detection application. The trained apple-detection model weights (.pt) were first converted to ONNX format (.onnx), then quantized to float16 precision and transformed into NCNN format (.param and .bin). Finally, the application was developed using Android Studio.

**Figure 9:**
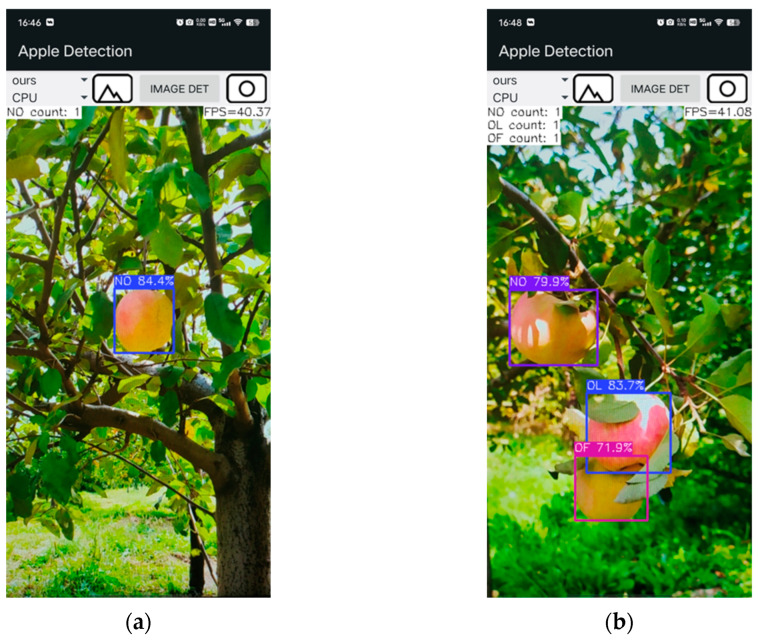
User interface of the developed Android application. (**a**) Single-category View; (**b**) Multi-category View.

The application captures images from the device’s camera, draws bounding boxes around the apples, and displays the subcategory of each apple, as shown in Figure 9. Additionally, it provides real-time feedback on detection speed and the count of different apple subcategories in the current interface.

### 2.5. Evaluation Metrics

To comprehensively assess the model’s performance, we utilized the following metrics: precision (P), recall (R), mean Average Precision (mAP), parameter count, Floating Point Operations (FLOPs), model size, and Frames Per Second (FPS). P denotes the proportion of correctly identified apples of a specific subcategory among all apples classified as that subcategory, as given in Equation (13). R indicates the proportion of correctly identified apples of a specific subcategory out of the total number of actual apples of that subcategory in the data set, as expressed in Equation (14):

(13) $$P=\frac{TP}{TP+FP}$$

(14) $$R=\frac{TP}{TP+FN}$$

where TP is the number of positive samples correctly detected, FP is the number of samples incorrectly detected as positive, and FN is the number of positive samples missed. IoU is the ratio of the overlapping area between the model’s predicted bounding box and the ground truth bounding box to the total area covered by both boxes, as shown in Equation (15):

(15) $$IoU=\frac{A\cap B}{A\cup B}$$

where A denotes the predicted bounding box, and B denotes the ground truth bounding box. In this study, a predicted bounding box was considered likely to be a TP for one of the four apple subcategories if its IoU with the ground truth exceeded 0.5. Average Precision (AP) represents the average of precision values at all recall levels between 0 and 1, which corresponds to the area beneath the Precision-Recall (P-R) curve. It is given by Equation (16). mAP is the mean AP across all C (C = 4) classes, as given in Equation (17).

Parameter count indicates the total number of parameters within the model, as shown in Equation (18). FLOPs measure the efficiency of the model’s execution, denoting the amount of computation required during prediction. This metric is expressed in Equation (19) when ignoring the number of addition operations. Model size refers to the storage space occupied by the model. FPS evaluates the model’s prediction speed, representing the number of images processed per second.

(16) $$AP=\int_{0}^{1}P(R)dR$$

(17) $$mAP=\frac{1}{C}\sum_{c=1}^{C}AP_{c}$$

(18) $$Param=\sum (K^{2}\times C_{in}\times C_{out})$$

(19) $$FLOPs=\sum (H\times W\times K^{2}\times C_{in}\times C_{out})$$

### 2.6. Experimental Environment

The model was trained on a computer equipped with an Intel Xeon Gold 6226R CPU, 32 GB of RAM, and an NVIDIA GeForce RTX 3090 GPU. The software environment included Ubuntu 20.04, CUDA 11.4, cuDNN v8.8.0, Python 3.8.18, and PyTorch 1.12.1. The parameters for the training regimen were configured with the following specifications: a batch size of 64, the polynomial coefficient $\varepsilon_{1}$ was set to −0.3, Stochastic Gradient Descent (SGD) optimizer with an initial learning rate of 0.01, a momentum of 0.937, and a weight decay of 0.0005. The model was trained from scratch for a maximum of 300 epochs.

The computer used to test the models ran Ubuntu 18.04 and featured an Intel Core i7-8700K CPU, an NVIDIA GeForce RTX 2080 Ti GPU, and 16 GB of RAM.

## 3. Results and Discussion

### 3.1. Performance of the Improved YOLOv8n Model

**Figure 10:**
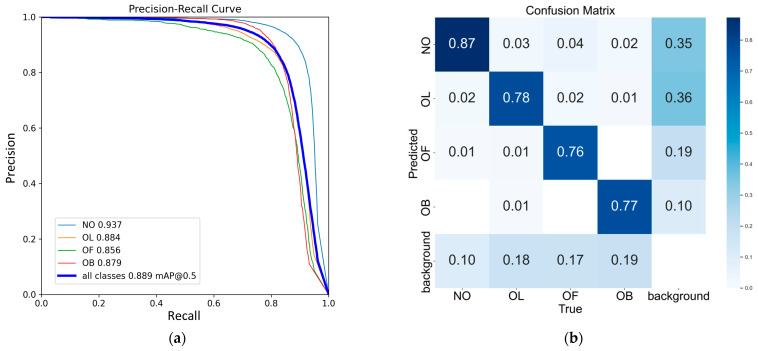
P-R curves and confusion matrix of improved YOLOv8n test results. (**a**) P-R curve; (**b**) Confusion matrix.

The precision-recall (P-R) curves of the improved YOLOv8n model for four distinct occluded apple categories in the test set are depicted in Figure 10a. The model demonstrated superior AP in identifying NO, with comparatively lower APs for OL, OF, and OB. Despite this, the AP for all categories exceeded 85%, resulting in an overall mAP of 88.9%. The confusion matrix in Figure 10b shows the highest recall of 87% for ON, followed by 78%, 76%, and 77% for OL, OF, and OB, respectively. The primary source of confusion occurred between apples and background elements, leading to missed detections or false positives. These results indicate that the model consistently maintains high accuracy in detecting each apple subcategory, ensuring reliable performance across different occlusion scenarios.

### 3.2. Comparison with Other Object-Detection Models

To further validate the advantages of the improved YOLOv8n apple-detection model, comparative evaluations were conducted with eight prevalent conventional object-detection models (Faster R-CNN [10], RetinaNet [11], SSD [9], YOLOv5l, YOLOv6-L [12], YOLOv7 [15], YOLOv8l, and YOLOv9-C [14]) and eight lightweight models (RT-DETR-R18 [8], RT-DETR-R34 [8], YOLOv5n, YOLOv6-N [12], YOLOv7-tiny [15], YOLOv8n, YOLOv9-T [14], and YOLOv11n [16]). For fairness, each model was trained and tested under identical conditions using the same apple data set as the improved YOLOv8n model.

**Figure 11:**
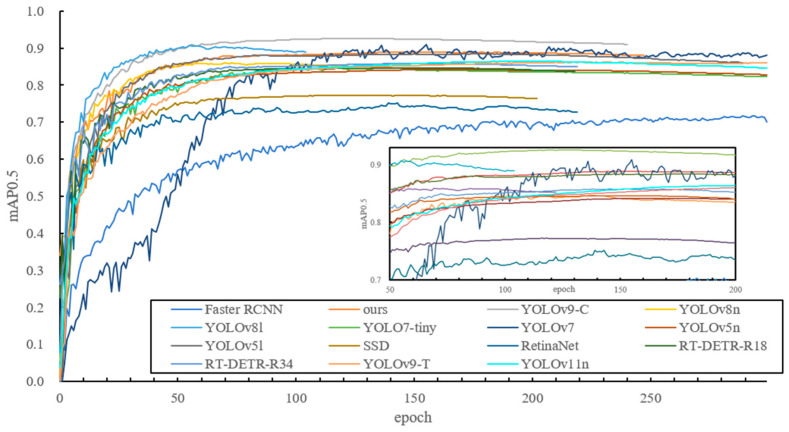
MAP curves for different models.

The mAP curves for the different models are depicted in Figure 11. During the first 80 iterations, the mAP increased rapidly for all models, then slowed down, stabilizing around epoch 300. Early stopping was used to prevent overfitting in the improved YOLOv8n model and several others. The mAP progression indicates that, although the improved YOLOv8n model did not experience the fastest mAP increase in the early epochs, it outperformed other lightweight models after 150 epochs.

The performance comparison of the improved YOLOv8n model with eight conventional object-detection models is presented in Table 2. While the YOLOv9-C model achieved the highest mAP, the improved YOLOv8n model reached a detection speed of 220 FPS, which is 185 FPS higher. Additionally, the improved YOLOv8n model had 99.37% fewer parameters, a 99.12% smaller model size, and 96.68% fewer FLOPs compared to YOLOv9-C. The improved YOLOv8n model’s mAP was 88.90%, slightly lower than that of YOLOv5l, YOLOv6-L, YOLOv7, and YOLOv8l, but it significantly outperformed these models in detection speed by 156 FPS, 176 FPS, 149 FPS, and 166 FPS, respectively. Moreover, the FLOPs of the improved YOLOv8n model were 92.66%, 94.76%, 92.37%, and 95.21% lower than those of YOLOv5l, YOLOv6-L, YOLOv7, and YOLOv8l, respectively. The model size and parameter count were also reduced by over 98%. Additionally, the improved YOLOv8n model’s mAP surpassed that of Faster R-CNN, RetinaNet, and SSD by 17.16%, 13.74%, and 11.64%, respectively.

As shown in Table 3, the performance comparison of the improved YOLOv8n model with eight lightweight object-detection models shows that the Transformer-based RT-DETR-R18 achieved a mAP of 84.65%, only marginally surpassing the CNN-based YOLOv5n by 0.45%. Despite this slight advantage in accuracy, RT-DETR-R18 exhibited slower detection speeds compared to all other lightweight CNN-based models. YOLOv5n exhibited lower FLOPs relative to all other lightweight models. However, its relatively low detection accuracy hinders its application in detecting apples within complex orchard environments. YOLOv8n offered a well-balanced compromise between detection accuracy and speed. It achieved a mAP that was 1.80% higher than YOLOv5n, with just a 7 FPS reduction in detection speed. In contrast, YOLOv11n showed a significant decrease in detection speed—12 FPS slower than YOLOv8n—with only a minor improvement in detection accuracy, gaining just 0.40% over YOLOv8n. Therefore, selecting YOLOv8n as the baseline model in this study aligns with our aim to achieve faster and more accurate detection.

The improved YOLOv8n model demonstrated a significant advantage in AP for OL and OB over other lightweight models. Moreover, it excelled across all key performance metrics by achieving the highest mAP, maintaining the smallest model size, minimizing the parameter count, and delivering the highest FPS among all lightweight models. Specifically, the improved YOLOv8n outperformed RT-DETR-R18, RT-DETR-R34, YOLOv5n, YOLOv6-N, YOLOv7-tiny, YOLOv8n, YOLOv9-T, and YOLOv11n, with mAP increases of 4.30%, 3.10%, 4.70%, 3.60%, 4.20%, 2.90%, 2.70%, and 2.50%, respectively. The model sizes were reduced by 99.72%, 99.82%, 76.92%, 91.35%, 92.68%, 85.48%, 85.25%, and 83.64%, respectively, while the parameter count decreased by 98.41%, 98.98%, 81.82%, 93.09%, 94.68%, 89.37%, 87.79%, and 87.60%, respectively. Furthermore, the detection speed increased by 177 FPS, 182 FPS, 44 FPS, 122 FPS, 93 FPS, 51 FPS, 167 FPS, and 63 FPS, respectively. These results indicate that the improved YOLOv8n model is better suited for deployment on picking robot devices with limited computational resources.

### 3.3. Qualitative Assessment

**Figure 12:**
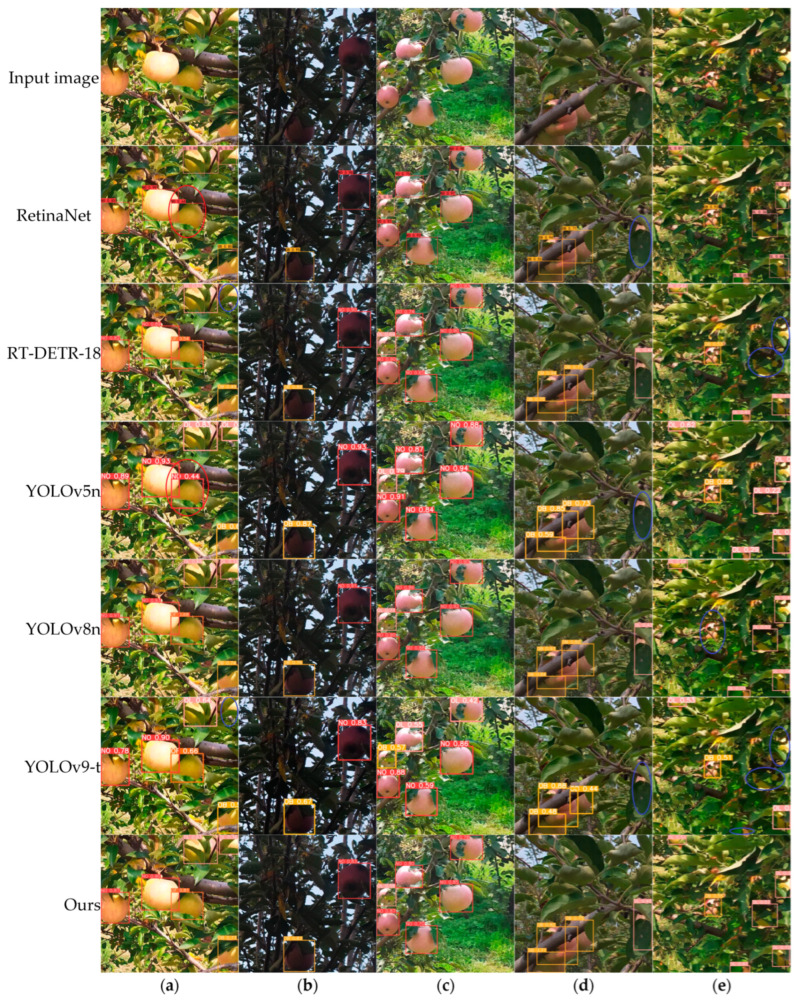
Comparison of detection results of different models under different lighting conditions and degrees of occlusion. (**a**) Direct light; (**b**) Low light; (**c**) Mild occlusion; (**d**) Moderate occlusion; (**e**) Severe occlusion. Marked blue ellipses indicate missed apples, and red ellipses indicate misclassified apples.

To provide a more intuitive evaluation of the detection performance of different object-detection models in complex orchard environments, images from various scenarios were chosen at random from the test data set for analysis. Figure 12 shows the detection results for apples under different lighting conditions and occlusion levels using several of the models evaluated in this study. Notably, the other models not depicted in the figure successfully detected all apples shown.

It can be observed that under low light conditions, all models correctly detected the target apples. In direct light, however, the RetinaNet and YOLOv5n models misclassified an OF apple as NO, while the RT-DETR-R18 and YOLOv9-T models missed detecting one apple. With mild occlusion, all models maintained accurate detection without errors. Under moderate occlusion, the RetinaNet, YOLOv5n, and YOLOv9-T models missed detecting one apple each. In severe occlusion scenarios, the RT-DETR-R18, YOLOv6n, YOLOv8n, and YOLOv9-T models missed two, one, one, and three detections, respectively.

The improved YOLOv8n model correctly detected all apples in Figure 12 and performed well in detecting all apple subcategories. These results highlight the effectiveness of incorporating SCC attention, PCMR, and PolyLoss into the YOLOv8n model, as well as utilizing features from the second and third pyramid levels of the backbone for multi-scale information fusion. The improved YOLOv8n model outperforms the original YOLOv8n model, demonstrating robust performance in apple detection across various scenarios.

### 3.4. Model Visualization

**Figure 13:**
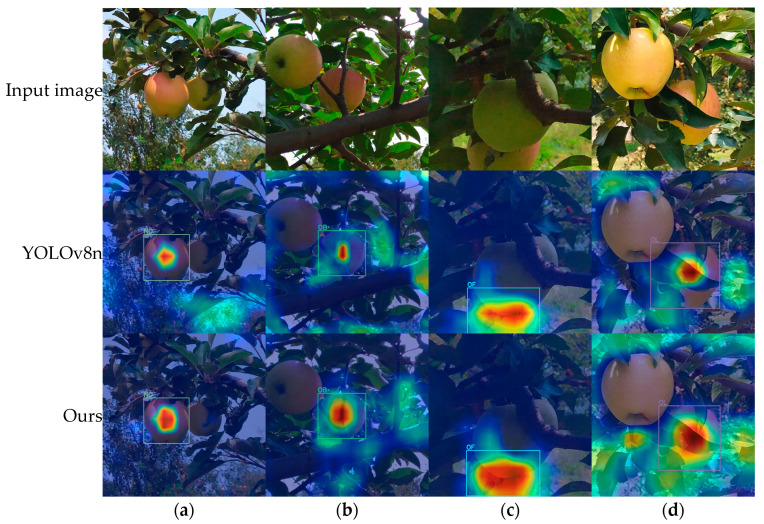
Comparison of heat maps before and after yolov8n model improvement. (**a**) NO; (**b**) OB; (**c**) OF; (**d**) OL. Regions highlighted in red indicate areas deemed important by the detection model, with darker red representing higher importance. Conversely, yellow and green hues denote areas of lesser significance, while blue indicates regions that contribute minimally to the model's decision-making process.

Grad-CAM [38] uses gradients to determine the importance of different regions in an image for the prediction result, thereby generating visualizations. Regions considered important by the detection model are highlighted in red, with deeper red indicating greater importance, while yellow and blue represent lesser contributions and almost no contribution, respectively. Figure 13 shows heat maps for different apple subcategories, illustrating that the model primarily concentrates on the central region of non-occluded apples. In contrast, it focuses on the junction between the apple and the occluder when detecting occluded apples. Additionally, Figure 13 illustrates that the improved YOLOv8n model focuses on more target regions compared to the original YOLOv8n model, enabling it to learn and utilize more discriminative features.

### 3.5. Ablation Experiments

To evaluate the effectiveness of the four improvements, ablation experiments were conducted on the improved YOLOv8n model. The results are presented in Table 4. Introducing SCC attention effectively suppressed background interference and enhanced the model’s feature extraction capability, leading to a 1.10% enhancement in mAP, accompanied by a mere 0.33% increase in parameter count and a decrease of 8 FPS in detection speed. Using PCMR as the feature fusion module reduced unnecessary convolution operations, resulting in lower parameter count, smaller model size, and fewer FLOPs. Employing PolyLoss increased mAP with minimal additional overhead. Furthermore, replacing the original three-layer feature fusion (p3, p4, p5) with a two-layer feature fusion (p2, p3) simplified the neck network, reducing parameter count and model size, while increasing mAP and improving detection speed. Collectively, these experimental results demonstrate that incorporating SCC attention and PolyLoss enhances the model’s accuracy, while adopting PCMR and a lightweight neck network significantly increases detection speed and decreases parameter count and model size.

### 3.6. Comparative Experiments with Different Attention Mechanisms

To evaluate the effectiveness of our SCC attention module, we compared it with other attention mechanisms: shuffle attention (SA) [39], squeeze-and-excitation (SE) [40], convolutional block attention module (CBAM) [41], and coordinate attention (CA) [35]. The experimental results are presented in Table 5.

Table 5 shows that integrating an attention module after the SPPF led to a slight increase in model size, a decrease in detection speed, but an improvement in mAP on the test set. Notably, our SCC attention module achieved a more significant improvement in detection accuracy, surpassing SA, SE, CBAM, and CA by 0.80%, 0.70%, 0.40%, and 0.30%, respectively. This superior performance can be attributed to the SCC attention module’s ability to more effectively focus the model on apples, enabling the extraction of more discriminative features. The results demonstrate that our SCC attention module outperforms mainstream attention mechanisms in terms of improving detection accuracy.

### 3.7. Comparative Experiments with Different Classification Losses

To evaluate the effectiveness of PolyLoss as a classification loss function for YOLOv8n, it was compared with several other classification loss functions: BCE Loss [42], Focal Loss [11], and Slide Loss [43]. The experimental results, shown in Table 6, reveal that PolyLoss achieved a higher detection accuracy of 86.90% compared to the other classification losses. Notably, BCE Loss outperformed Focal Loss and Slide Loss in terms of accuracy. This can be attributed to the inclusion of Distribution Focal Loss (DFL) in the regression loss of YOLOv8n, which addresses class imbalance and focuses on difficult-to-classify samples, compensating for the disadvantage of using BCE Loss as the classification loss.

PolyLoss is more flexible than BCE Loss (which is a special case of PolyLoss) and can be customized for specific data sets. For our data set, the optimal polynomial coefficient, $\varepsilon_{1}$, for PolyLoss was found to be −0.3. A negative $\varepsilon_{1}$ reduces prediction confidence, achieving effects similar to label smoothing and confidence penalties [37], which helps the YOLOv8 model achieve higher detection accuracy.

### 3.8. Multi-Scale Feature Fusion Optimization Experiment

In this study, we investigated the impact of various feature combinations on apple-detection performance by extracting features from the second (P2), third (P3), fourth (P4), fifth (P5), and sixth (P6) layers of our backbone architecture. This allowed us to assess how these combinations affect prediction performance. We utilized a retrained multi-scale feature fusion network for predictions on the test set. The results, presented in Table 7, show that the combination of feature layers P2 and P3 yielded the most favorable overall performance. Specifically, its mAP was only 0.40% lower than that of the highest-performing three-layer combination (P2, P3, P4). Moreover, it offered a significant advantage in terms of efficiency: a 43 FPS increase in detection speed, a 66.32% decrease in parameter count, a 60.87% reduction in model size, and a 21.78% decrease in FLOPs.

The likely reason for this is that higher layers (P4, P5, and P6) have larger receptive fields, which makes it harder to capture sufficient discriminative information for small objects, leading to missed or incorrect detections. In contrast, features from P2 and P3 retain more discriminative information about small apples, providing most of the useful information from the combination of P2, P3, and P4 features.

The experimental results demonstrate that using features from the P2 and P3 layers as inputs to the neck network achieves an optimal balance between mAP and detection speed, the two most critical metrics for object-detection performance.

### 3.9. Android App Results

**Figure 14:**
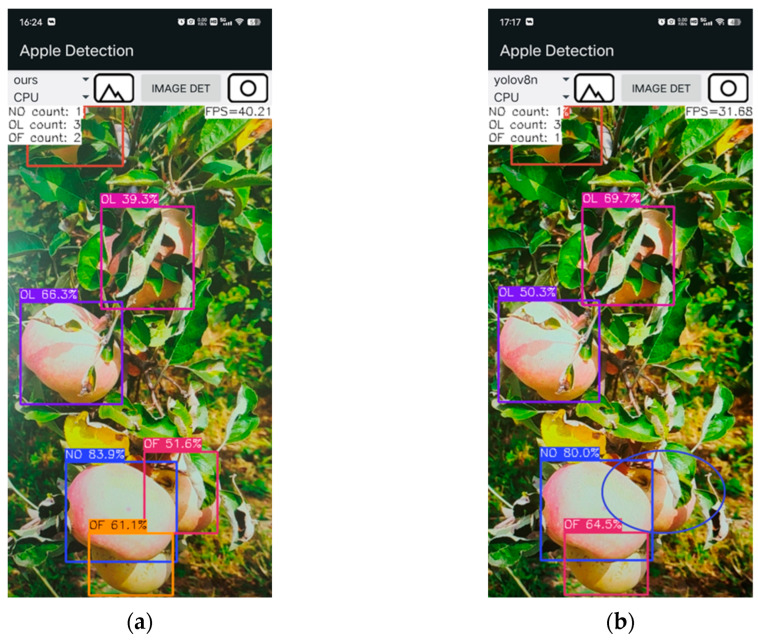
Comparison of the improved YOLOv8n and the original YOLOv8n in application deployment. (**a**) Improved YOLOv8n; (**b**) Original YOLOv8n. Marked blue ellipses indicate missed apples.

The apple-detection application developed in this study achieved a detection speed of 40 FPS on an iQOO Neo6 SE smartphone using the improved YOLOv8n model, compared to 32 FPS with the original YOLOv8n model, as shown in Figure 14. Moreover, Figure 14 illustrates that the improved YOLOv8n model correctly detected all apples in the image, whereas the original YOLOv8n model missed one. These results suggest that the improved YOLOv8n model significantly improves both the speed and accuracy of apple detection.

To evaluate the performance of the improved YOLOv8n model more comprehensively, we compared it with the original YOLOv8n model in terms of battery consumption. In the comparison experiment, each model was tested five times. In each test, the iQOO Neo6 SE smartphone was charged to 100%, and the model’s app started to run after 10 min of idle placement. Each experiment lasted for 60 min. The experimental results, shown in Table 8, indicate that the battery-consumption rate of the improved YOLOv8n model is slightly lower than that of the original model. This is primarily due to the improved YOLOv8n model reducing redundant computations, parameter count, and memory accesses, which helps maintain a higher detection speed without accelerating battery consumption.

### 3.10. Limitations and Future Work

**Figure 15:**
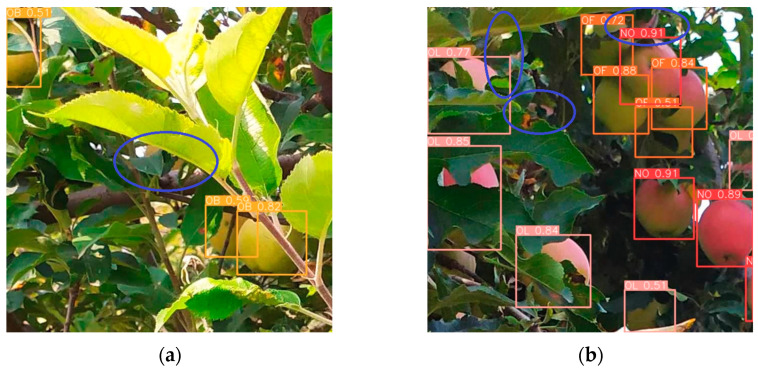
Examples of missed detection. (**a**) Green apples; (**b**) Slightly red apples. Marked blue ellipses indicate missed apples.

Figure 15 illustrates instances where the improved YOLOv8n model missed detecting apples, indicated by blue ellipses.

The potential causes for these missed detections include: (1) Excessive occlusion of the apples results in fewer visible pixels, which makes it challenging for the model to extract sufficient and effective discriminative features, thereby leading to missed detections. (2) As illustrated in Figure 15a, an apple that is mostly occluded by leaves and whose color closely matches the background increases the complexity of the detection task.

To mitigate the limitations of the improved YOLOv8n model, the following measures can be taken: (a) Collect additional data sets that include instances prone to causing missed or incorrect detections. This will allow the model to learn to extract features more effectively from apples that are difficult to recognize. (b) Utilize advanced data augmentation techniques, such as adding leaf textures to apple locations in the images of the training set or using Generative Adversarial Networks (GANs) to generate realistic occlusions, enhancing the model’s ability to recognize occluded apples. (c) Investigate alternative feature-extraction methods that maintain detection speed while minimizing the loss of critical features, thereby improving the model’s accuracy.

In the future, we plan to integrate the improved YOLOv8n model into an apple-harvesting robot system to rigorously validate its reliability and performance in real-world conditions.

## 4. Conclusions

In this study, we proposed an improved object-detection model based on YOLOv8n, achieving rapid and accurate detection of apples. We incorporated the SCC attention module to improve detection performance, replaced the C2f module in the neck of YOLOv8n with the PCMR module to achieve model lightweighting, and utilized features from the P2 and P3 layers for multi-scale information fusion. Additionally, we employed PolyLoss to better adapt the model to the apple data set. Experimental results demonstrated that the improved YOLOv8n model enhanced lightweight properties, detection speed, and mAP compared to the original YOLOv8n model. Specifically, the parameter counts and FLOPs were reduced by 89.36% and 2.47%, respectively, the detection speed increased by 30.55% to 220 FPS, and mAP improved by 2.90% to 88.9%. The AP values for NO, OL, OF, and OB apples were 93.70%, 88.40%, 85.60%, and 87.90%, respectively. In comparison with Faster R-CNN, RetinaNet, SSD, RT-DETR-R18, RT-DETR-R34, YOLOv5n, YOLOv6-N, YOLOv7-tiny, YOLOv8n, YOLOv9-T, and YOLOv11n object-detection models, the improved YOLOv8n model exhibited superior performance in terms of mAP and detection speed. Furthermore, the improved YOLOv8n model was quantized to float16 and developed into an apple-detection application using Android Studio. This application enables real-time apple detection in complex orchard environments on mobile devices. Together, the improved YOLOv8n model demonstrates higher detection accuracy and speed while maintaining superior lightweight properties, making it especially suitable for deployment on resource-constrained devices requiring real-time apple detection.

However, the improved YOLOv8n model proposed in this study can currently only determine the position of the apple in the image, with precise 3D spatial coordinates remaining to be determined for its application in apple-picking robotic systems. To address this, we plan to integrate the prediction results of the improved YOLOv8n model with depth information from a stereo camera, enabling effective apple detection and localization. Given that stereo cameras require significant computational resources to calculate depth through parallax, ensuring real-time performance upon integration with the improved YOLOv8n model may necessitate higher-performance hardware. Further research will be required in the future to optimize algorithms and enhance hardware acceleration.

## Figures and Tables

**Table 1:** Distribution of data set.

Dataset Category	Image	NO	OL	OF	OB	Augmented
Training set	2310	11,502	6767	2518	5631	√
Validation set	121	641	322	171	429	×
Test set	243	1191	870	343	705	×

**Table 2:** Performance comparison of the improved model with conventional object-detection models.

Models	AP NO (%)	OL (%)	OF (%)	OB (%)	mAP0.5 (%)	Params (M)	Model Size (MB)	FLOPs (G)	FPS
SSD	85.40	76.20	77.20	70.30	77.30	26.29	96.60	282.20	44.77
RetinaNet	83.50	71.50	76.90	68.70	75.20	37.97	145.90	191.10	43.05
Faster_RCNN	84.50	68.50	68.20	65.70	71.70	137.10	113.50	402.40	20.00
YOLOv5l	96.30	89.80	88.90	90.60	91.40	46.12	92.90	107.70	64.10
YOLOv6-L	96.20	88.00	90.10	88.00	90.60	59.54	119.60	150.70	43.40
YOLOv7	96.30	88.60	87.90	90.80	90.90	36.50	74.80	103.50	71.05
YOLOv8l	95.70	88.50	89.60	89.00	90.70	43.61	87.70	164.80	53.48
YOLOv9-C	97.00	91.50	91.70	90.20	92.60	50.97	102.80	238.00	34.41
ours	93.70	88.40	85.60	87.90	88.90	0.32	0.90	7.90	219.71

**Table 3:** Performance comparison of different lightweight models.

**Models**	**AP** **NO** **(%)**	**OL** **(%)**	**OF** **(%)**	**OB** **(%)**	**mAP0.5** **(%)**	**Params** **(M)**	**Model Size (MB)**	**FLOPs** **(G)**	**FPS**
RT-DETR-R18	95.40	80.90	87.60	74.60	84.60	20.09	322.30	60.40	42.32
RT-DETR-R34	95.20	82.50	88.70	76.80	85.80	31.32	502.30	92.40	37.27
YOLOv5n	91.70	82.20	80.90	82.00	84.20	1.76	3.90	4.10	175.44
YOLOv6-N	92.20	83.40	84.70	80.80	85.30	4.63	10.40	11.40	98.00
YOLOv7-tiny	92.00	83.40	83.90	79.50	84.70	6.02	12.30	13.10	126.28
YOLOv8n	92.70	84.80	85.10	81.50	86.00	3.01	6.20	8.10	168.29
YOLOv9-T	93.20	82.90	86.70	81.90	86.20	2.62	6.10	10.70	52.36
YOLOv11n	93.20	84.00	86.10	82.20	86.40	2.58	5.50	6.30	156.25
ours	93.70	88.40	85.60	87.90	88.90	0.32	0.90	7.90	219.71

**Table 4:** Results of ablation experiments.

**S**	**PC**	**Po**	**p2,p3**	**P** **(%)**	**R** **(%)**	**AP** **NO** **(%)**	**OL** **(%)**	**OF** **(%)**	**OB** **(%)**	**mAP0.5** **(%)**	**Params** **(M)**	**Model Size (MB)**	**FLOPs** **(G)**	**FPS**
×	×	×	×	92.10	77.00	92.70	84.80	85.10	81.50	86.00	3.01	6.20	8.10	168.29
√	×	×	×	94.00	76.60	93.60	85.10	87.10	82.60	87.10	3.02	6.30	8.10	160.31
×	√	×	×	92.00	77.00	91.30	83.60	85.00	83.10	85.80	2.86	5.90	7.80	177.54
×	×	√	×	92.10	77.30	92.60	85.80	86.70	82.40	86.90	3.01	6.20	8.10	167.92
×	×	×	√	88.90	78.60	92.30	86.40	85.30	83.50	86.90	0.32	0.90	8.00	217.8
√	√	×	×	93.70	76.30	93.40	84.70	85.70	83.50	86.80	2.87	6.00	7.80	170.36
√	√	√	×	92.30	78.50	94.00	85.60	86.20	84.70	87.60	2.87	6.00	7.80	170.07
√	√	√	√	89.00	81.50	93.70	88.40	85.60	87.90	88.90	0.32	0.90	7.90	219.71

S, PC, and Po represent SCC, PCMR, and PolyLoss, respectively.

**Table 5:** Comparative results of different attention modules.

**Models**	**AP** **NO** **(%)**	**OL** **(%)**	**OF** **(%)**	**OB** **(%)**	**mAP0.5** **(%)**	**Params** **(M)**	**Model Size (MB)**	**FLOPs** **(G)**	**FPS**
YOLOv8n	92.70	84.80	85.10	81.50	86.00	3.01	6.20	8.10	168.29
YOLOv8n + SA	92.10	84.90	85.20	82.90	86.30	3.01	6.30	8.10	164.50
YOLOv8n + SE	92.60	83.50	86.30	83.30	86.40	3.01	6.30	8.10	166.00
YOLOv8n + CBAM	92.70	85.40	85.30	83.50	86.70	3.01	6.30	8.10	162.93
YOLOv8n + CA	92.60	85.50	85.20	83.90	86.80	3.01	6.30	8.10	162.65
YOLOv8n + SCC	93.60	85.10	87.10	82.60	87.10	3.02	6.30	8.10	160.31

**Table 6:** Comparison results of different classification losses.

**Loss**	**P** **(%)**	**R** **(%)**	**AP** **NO** **(%)**	**OL** **(%)**	**OF** **(%)**	**OB** **(%)**	**mAP0.5** **(%)**
BCE	92.10	770	92.70	84.80	85.10	81.50	86.00
Focal	90.70	72.70	85.30	77.80	78.90	80.00	80.50
Slide	90.80	77.80	92.40	84.70	85.50	81.10	85.90
PolyLoss	92.10	77.30	92.60	85.80	86.70	82.40	86.90

**Table 7:** Comparative results of multi-scale feature fusion experiments.

**Methods**	**P** **(%)**	**R** **(%)**	**AP** **NO** **(%)**	**OL** **(%)**	**OF** **(%)**	**OB** **(%)**	**mAP0.5** **(%)**	**Params** **(M)**	**Model Size (MB)**	**FLOPs** **(G)**	**FPS**
A	77.20	58.80	73.80	65.70	50.20	66.10	64.00	3.58	7.30	3.70	181.27
B	89.40	76.60	92.10	85.80	81.30	81.60	85.20	4.30	8.80	5.10	160.57
C	85.00	80.00	91.10	85.30	81.10	81.70	84.80	2.55	5.30	5.00	199.78
D	92.30	78.50	94.00	85.60	86.20	84.70	87.60	2.87	6.00	7.80	170.07
E	87.00	79.90	94.10	86.40	84.40	84.50	87.40	0.94	2.10	6.10	205.10
F	89.40	82.90	94.50	87.50	87.30	87.70	89.30	0.95	2.30	10.10	176.65
G	89.00	81.50	93.70	88.40	85.60	87.90	88.90	0.32	0.90	7.90	219.71

A represents layers p5 and p6; B represents layers p4, p5, and p6; C represents layers p4 and p5; D represents layers p3, p4, and p5; E represents layers p3 and p4; F represents layers p2, p3, and p4; G represents layers p2 and p3.

**Table 8:** Comparison of battery-level depletion rates.

**Models**	**Trial 1** **(%/MIN)**	**Trial 2** **(%/MIN)**	**Trial 3** **(%/MIN)**	**Trial 4** **(%/MIN)**	**Trial 5** **(%/MIN)**	**Average** **(%/MIN)**
YOLOv8n	−0.283	−0.300	−0.300	−0.300	−0.300	−0.296
ours	−0.300	−0.300	−0.283	−0.283	−0.300	−0.293

## Data Availability

Dataset available on request from the authors. The raw data supporting the conclusions of this article will be made available by the authors on request.
